# Chemical genetics approach to restoring p27Kip1 reveals novel compounds with antiproliferative activity in prostate cancer cells

**DOI:** 10.1186/1741-7007-8-153

**Published:** 2010-12-23

**Authors:** Elizabeth Rico-Bautista, Chih-Cheng Yang, Lifang Lu, Gregory P Roth, Dieter A Wolf

**Affiliations:** 1 Signal Transduction Program, Sanford-Burnham Medical Research Institute, La Jolla, CA 92037, USA; 2 GE Healthcare, Life Sciences, Shanghai 201203, China; 3 Sanford-Burnham Medical Research Institute at Lake Nona, Orlando, FL 32827, USA

## Abstract

**Background:**

The cyclin-dependent kinase (CDK) inhibitor p27^Kip1 ^is downregulated in a majority of human cancers due to ectopic proteolysis by the ubiquitin-proteasome pathway. The expression of p27 is subject to multiple mechanisms of control involving several transcription factors, kinase pathways and at least three different ubiquitin ligases (SCF^SKP2^, KPC, Pirh2), which regulate p27 transcription, translation, protein stability and subcellular localization. Using a chemical genetics approach, we have asked whether this control network can be modulated by small molecules such that p27 protein expression is restored in cancer cells.

**Results:**

We developed a cell-based assay for measuring the levels of endogenous nuclear p27 in a high throughput screening format employing LNCaP prostate cancer cells engineered to overexpress SKP2. The assay platform was optimized to Z' factors of 0.48 - 0.6 and piloted by screening a total of 7368 chemical compounds. During the course of this work, we discovered two small molecules of previously unknown biological activity, SMIP001 and SMIP004, which increase the nuclear level of p27 at low micromolar concentrations. SMIPs (small molecule inhibitors of p27 depletion) also upregulate p21^Cip1^, inhibit cellular CDK2 activity, induce G1 delay, inhibit colony formation in soft agar and exhibit preferential cytotoxicity in LNCaP cells relative to normal human fibroblasts. Unlike SMIP001, SMIP004 was found to downregulate SKP2 and to stabilize p27, although neither SMIP is a proteasome inhibitor. Whereas the screening endpoint - nuclear p27 - was robustly modulated by the compounds, SMIP-mediated cell cycle arrest and apoptosis were not strictly dependent on p27 and p21 - a finding that is explained by parallel inhibitory effects of SMIPs on positive cell cycle regulators, including cyclins E and A, and CDK4.

**Conclusions:**

Our data provide proof-of-principle that the screening platform we developed, using endogenous nuclear p27 as an endpoint, presents an effective means of identifying bioactive molecules with cancer selective antiproliferative activity. This approach, when applied to larger and more diverse sets of compounds with refined drug-like properties, bears the potential of revealing both unknown cellular pathways globally impinging on p27 and novel leads for chemotherapeutics targeting a prominent molecular defect of human cancers.

## Background

p27 is a cyclin-dependent kinase (CDK) inhibitor (CKI) that controls cell proliferation, cell motility and apoptosis [1]. It regulates the progression of cells from G1 to S phase by binding and inhibiting the cyclin E-CDK2 complex. A plethora of evidence has implicated downregulation of p27 in prevalent human carcinomas [1]. For example, downregulation of p27 is among the most frequent non-genetic molecular alterations in prostate cancer (PCa) [2]. In this disease, low p27 expression is correlated with a number of prognostic morphological features [3] and with decreased survival [4]. In contrast, ectopic expression of p27 can inhibit cell cycle progression in a human PCa cell line [5], suppress astrocytoma growth in nude mice [6] and induce the death of breast cancer cells [7]. Based on these findings, p27 has been denoted as a tumour suppressor.

The regulation of p27 during the cell cycle is very complex [1]. It involves regulation at the level of transcription, messenger (m)RNA translation [8] and protein stability. The distribution among different cyclin-CDK complexes [9], its sub-cellular localization [10] as well as phosphorylation of several residues in p27 are important mechanisms of control [11-13]. p27 levels are high in quiescent cells and decrease rapidly upon mitogenic stimulation. However, the cell cycle-dependent variations in p27 levels are not reflected by similar changes in p27 mRNA [8,14]. Unlike traditional tumour suppressor genes, the p27 gene rarely undergoes homozygous inactivation in cancer cells [15-17], a finding that points towards alternative mechanisms of p27 inactivation. Many aggressive cancers display decreased p27 protein levels in the presence of high p27 mRNA [18,19], suggesting that p27 depletion is primarily a result of ectopic proteolysis.

The p27 protein accumulates in cells when the ubiquitin proteasome system (UPS) is inhibited [20]. This system employs a cascade of enzymatic reactions that covalently attach a ubiquitin chain to a substrate protein [21], leading to the recognition by the proteasome for degradation. Biochemical studies identified SCF^SKP2^, an ubiquitin ligase complex that mediates phosphorylation-dependent p27 ubiquitylation *in vitro *[13,22]. Two other enzymes, KPC and PIRH2, have been also been implicated as E3s for p27 [23-25]. Whereas SCF^SKP2 ^mediates the degradation of nuclear p27 throughout S phase and G2, KPC targets cytoplasmic p27 upon cell cycle entry from G0 [23,25]; PIRH2 instead targets nuclear and cytoplasmic p27 [24].

Considerable evidence suggests, however, that SKP2 is the prominent regulator of p27 levels in cancer cells [26]. SKP2 overexpression is frequent in human carcinomas devoid of p27 [27-32]. In addition, our own data have shown that SKP2 overexpression in LNCaP prostate cancer cells is sufficient to direct p27 ubiquitylation and degradation [33]. Furthermore, transgenic expression of SKP2 in the mouse prostate causes low-grade prostate carcinomas that coincide with p27 downregulation [32]. Conversely, RNA interference (RNAi)-mediated knockdown of SKP2 expression inhibits tumour growth in a mouse transplant model [34]. These findings validated p27 degradation pathways as promising cancer drug targets [35].

The complexity of p27 regulation presents considerable challenges to drug development because of the potential for redundancies. Thus, it is not readily apparent which enzyme involved in p27 regulation should be targeted in order to effect sustained p27 accumulation in cancer cells. In addition, pharmacological agents active in an enzyme assay *in vitro *are not necessarily bioactive in intact cells. Rather than targeting a preselected component, we have developed a method for the up-front identification of compounds that are bioactive in restoring physiological levels of p27 in prostate cancer cells. Using a validated cell-based assay, two compounds, small molecule inhibitors of p27 depletions (SMIPs) 001 and 004, were identified, which had the desired activity. SMIPs restored p27 to physiological levels, inhibited CDK2 activity and caused cell cycle delay or apoptosis selectively in prostate cancer cells but not normal cells.

## Results

### Generation and validation of an LNCaP-derived screening cell line overexpressing SKP2

**Figure 1:**
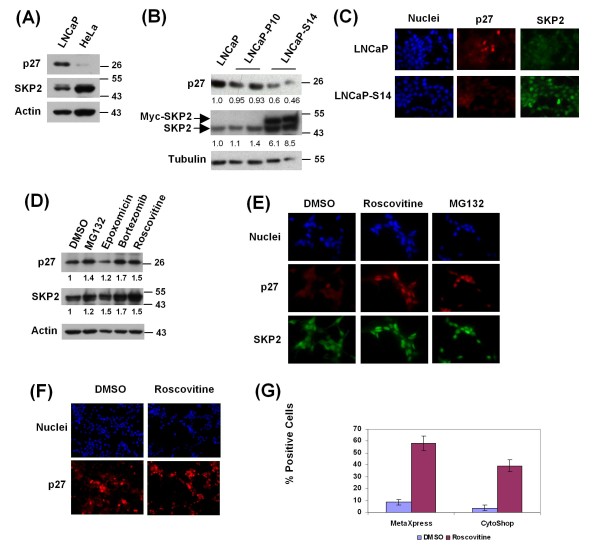
**Characterization of the LNCaP-S14 screening cell line**. (a) Basal levels of p27 and SKP2 were examined by immunoblotting in LNCaP and HeLa cells. (b) Parental LNCaP cells as well as cells stably transfected with empty vector (P10) or Myc-SKP2 (LNCaP-S14) were examined for the expression of p27 and SKP2 by immunoblotting. The numbers under each lane represent the tubulin normalized levels of p27 and SKP2 relative to untransfected LNCaP cells. (c) Basal levels of p27 and SKP2 in LNCaP and LNCaP-S14 were visualized by immunofluorescence. Images were taken using a Nikon Eclipse E600 microscope at 60× magnification. (d) The levels of p27 and SKP2 were analysed in total cell lysate from LNCaP-S14 cells treated with the proteasome inhibitors MG132 (20 μM), epoxomycin (1 μM) and bortezomib (100 nM) or the CDK inhibitor roscovitine (20 μM) for 18 h. The numbers under each lane represent actin normalized levels of p27 and SKP2 relative to DMSO treated cells. (e) p27 and SKP2 staining in cells treated with the indicated compounds for 18 h. (f) Cells seeded in a 384 well plate and treated with vehicle (dimethyl sulfoxide; DMSO) or roscovitine (20 μM) for 18 h were fixed and stained for p27. Images were obtained using the ImageXpressMicro automated microscope (Molecular Devices) at 20× magnification. (g) The graph represents the percentage of p27 positive cells +/- standard deviations in LNCaP-S14 cells treated with DMSO or roscovitine and analysed with two imaging platforms (MetaXpress and CytoShop) as described in the Methods section. Data are representative of three independent experiments.

Prostate cancers typically display an inverse correlation between the levels of p27 and SKP2 [29]. In contrast, the commonly used human prostate cancer cell line LNCaP, despite faithfully recapitulating many features of human prostate cancer [36], expresses relatively high levels of p27 but low levels of SKP2 when compared to HeLa cells (Figure 1a). In order to mimic the situation prevailing in primary prostate cancers, we created the LNCaP derivative cell line, LNCaP-S14, which stably overexpresses Myc-tagged SKP2 at six to eightfold excess over endogenous SKP2, a manoeuvre that led to maximal downregulation of p27 (Figure 1b). The same pattern was apparent by immunofluorescence staining; while nuclear levels of p27 are low in LNCaP-S14 cells, nuclear SKP2 levels are highly elevated (Figure 1c).

We also determined whether known inhibitors of p27 degradation could upregulate p27 in LNCaP-S14 cells. Upon normalization to the loading control actin, the proteasome inhibitors MG132, epoxomycin and bortezomib caused between 1.2 and 1.7-fold upregulation of p27, while the CDK inhibitor roscovitine led to 1.5-fold accumulation (Figure 1d). The induction of nuclear p27 by MG132 and roscovitine was also apparent by immunofluorescence (Figure 1e).

### Development of a high throughput screening assay to score the level of nuclear p27

The immunofluorescence assay used in Figure 1c and 1e was adapted to 384 well plate format. All parameters, including the number of cells to be seeded, fixation, blocking conditions, antibody concentrations and incubation times with compounds were extensively optimized using positive (proteasome and CDK inhibitors) and negative (dimethyl sulfoxide; DMSO) controls, resulting in the reliable protocol described in the Methods section. Representative images of LNCaP-S14 cells treated with DMSO or roscovitine and stained with the above protocol in 384 well plates are shown in Figure 1f.

In order to evaluate the performance of our assay to reliably measure the percentage of p27 positive cells in a cell population, we determined the Z'-factor for the positive control reagent roscovitine. The Z'-factor measures the variation and separation bands of an assay thereby providing a statistical measure of its quality. It takes into account the dynamic range and variability of the positive and negative control measurements [37]. We determined a Z'-factor of 0.48 using the ImageXpress Micro and MetaXpress software from Molecular Devices (CA, USA) and 0.60 using the Cell Lab IC100 and Cytoshop software from Beckman Coulter (USA). Both values are deemed appropriate for a successful screen [37]. Figure 1g shows the signal-to-background ratios using both imaging platforms.

### Primary screen

**Figure 2:**
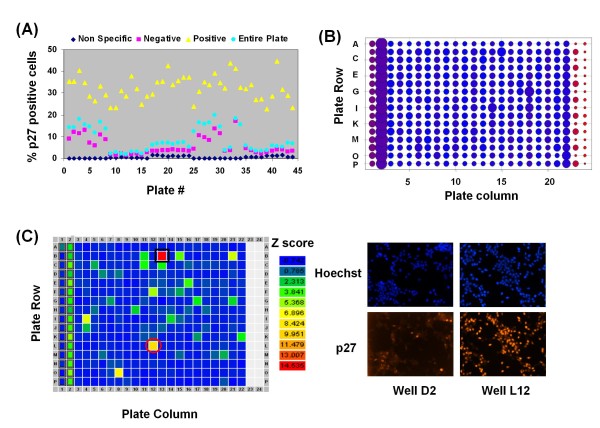
**Assay data from the primary screen**. (a) The graph represents the mean percentages of p27 positive cells detected across all wells in each of the 44 screening plates (blue circles). The data are further stratified by treatment: Red squares indicate the mean percentages obtained with the negative control (dimethyl sulfoxide; DMSO), yellow triangles indicate the mean percentages obtained with the positive control (roscovitine). Blue diamonds indicate the mean percentages obtained by staining without the primary p27 antibody (non-specific background staining). (b) Sum Z-scores. The heat map represents the sum of Z-scores from each well in 22 plates. DMSO (negative control) was added to column 1, roscovitine (positive control) to column 2, and in some cases to columns 22 and 23, and the non-specific staining was performed in columns 23 and 24. (c) Heat map of a representative screening plate showing potential hits based on their Z score. The first column on the plate contained the negative control DMSO, the second column contained the positive control agent roscovitine, and columns 23 and 24 were stained without the primary p27 antibody (non-specific background staining). The well marked with a black square was identified as containing a false positive compound, whereas the red circle indicates a potential hit. A representative micrograph from well L12 (potential hit) is shown to the right.

In order to assess the utility of the above protocol, we performed a pilot screen of 7368 compounds (derived from a variety of different compound libraries, see Methods) in duplicates for a total of 44 384-well plates. LNCaP-S14 cells were incubated with individual compounds (final concentration varied between ~5 and 35 uM depending on the molecular weights of the compounds) for 18 h, followed by fixation, staining, imaging (automatic microscopy) and analysis as described in Methods. We scored the percentage of cells positive for p27 in each well relative to the vehicle control (DMSO). Figure 2a shows the activity of positive control compounds (yellow triangles), the negative control compound DMSO (red squares), the non-specific staining (blue diamonds) and small molecules derived from compound libraries (blue circles) across all screening plates (44 plates) prior to normalization. Negative controls (DMSO) gave basal percentages of p27 positive cells ranging between 2% and 13% while the positive control roscovitine increased these populations up to 50%. As expected, the average of ~320 well measurements per plate - that is, the percentage of p27 positive cells in wells treated with individual compounds - was similar to the percentage of p27 positive cells in the negative control (DMSO), indicating that most compounds tested were inactive in causing p27 accumulation (Figure 2a).

Normalization of raw data for 7368 compounds was performed by calculating Z scores, which represent the number of standard deviations by which a signal for a given compound differed from the mean signal of the entire plate [38]. It excludes control measurements altogether under the assumption that most compounds are inactive and can serve as intrinsic controls. The summed Z scores for one set of duplicates (22 plates) showed clear separation between the positive control compound roscovitine (column 2) and the non-specific staining (cells treated with DMSO or stained with secondary antibody only, columns 23 and 24; Figure 2b). In addition, most summed Z scores had values similar to the negative controls (DMSO), indicating that we did not incur errors caused by plate position of compounds. This was further evaluated by visualizing every plate as a heat map of individual Z scores. As shown in the example of a single plate in Figure 2c, potential hits were randomly distributed over the plate (green and yellow positions). The analysis also identified several false-positive compounds with very high Z scores, namely known DNA binding compounds of red colour (doxorubicin and derivatives, crystal violet, homidium bromide, propidium iodine, pararosalinine pamoate).

### Results of the primary screen

**Figure 3:**
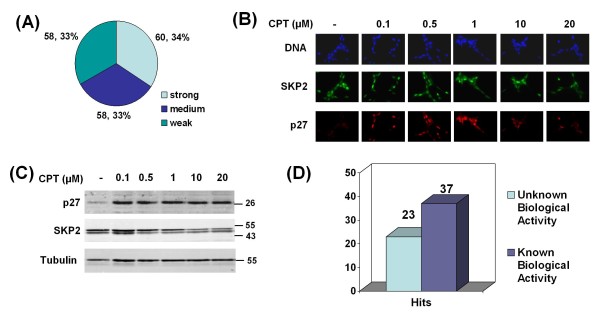
**Primary screening results**. (a) Summary of the primary screen in LNCaP-S14 cells. From 7368 compounds analysed in duplicates, 176 were positive in the primary screening. They were classified according to their Z-score as strong (Z > 5), medium (Z = 3-5) and weak (Z = 2-3). (b) Effect of increasing concentrations (18 h treatment) of the positive screening hit camptothecin (CPT) on p27 and SKP2 levels in LNCaP-S14 cells by regular immunofluorescence and immunoblotting (c). (d) 109 strong and medium compounds were selected for secondary screening. Of those 60 compounds were still positive.

In the initial primary screen, we identified 249 compounds that increased the percentage of p27 positive LNCaP-S14 cells. Upon manual examination of microscopic images, 21 duplicate compounds (occurring in more than one library), seven obvious false positives (DNA stains) and 45 compounds with low quality staining (few cells left in well, background, indiscriminate whole cell staining) were sorted out. This resulted in a list of 176 candidates (Additional File 1, Supplementary Table 1) with sixty compounds classified as strong (Z score > 5), 58 as medium (Z score 3-5) and the remainder as weak (Z score 2-3; Figure 3a). At a Z score of 3, a compound has a theoretical probability of 0.0013 of being a false positive hit.

**Table 1:** Cytotoxicity was analysed by MTT assay in LNCaP-S14 and IMR90 cells treated with increasing concentrations (0.01 - 40 μM) of the respective small molecule inhibitors of p27 depletion (SMIPs) for 72 h.

	LNCaP-S14	IMR90
MG132	0.379	0.445
Thapsigargin	0.0078	0.031
**SMIP001**	**4.63**	**> 20**
SMIP002	11.09	> 40
SMIP003	15.92	65.36
**SMIP004**	**1.09**	**> 20**
SMIP005	1.71	17.38
SMIP006	6.63	NT
SMIP009	> 40	> 40
SMIP011	> 40	~ 40
SMIP012	4.59	~ 10
SMIP013	18.15	~ 20
SMIP015	13.02	~ 20
SMIP016	14.63	4.41
SMIP017	23.73	> 40
SMIP018	> 40	> 40
SMIP019	NT	~ 40

NT: not tested.

The data in the table represents the IC50 (μM).

A number of experimental and clinically used chemotherapeutics such as camptothecin, thapsigargin, 17-N-allylamino-17-demethoxygeldanamycin, mitomycin C and etoposide were identified (Additional File 1, Supplementary Table 1). The PI3 kinase inhibitor Wortmannin also scored positive, a finding that is consistent with the known role of the PI3K/AKT pathway in the downregulation of p27 through SKP2 [39]. We further validated some of the known bioactive compounds by immunofluorescence staining and immunoblotting (Figure 3b and 3c; data not shown).

### Secondary screening

The one hundred and nine most potent compounds (Z ≥ 3) were selected for confirmation by secondary screening using the primary screening assay. After statistical analysis, 60 compounds were still considered positive hits (Figure 3d). It is well established that compounds with high activity in the primary screen will appear less potent in the secondary screen because of a statistical effect known as 'regression towards the mean' [38]. Thus, weaker hits often fail to validate in the secondary screen due to random measurement error. Additional counter screening for autofluorescence identified another false-positive (pyrvinium pamoate).

Thirty-seven of the 60 validated compounds had known biological activity (for example, inhibitors of protein synthesis and topoisomerase activity, antibiotics and antihelmintics). Of the remaining 23 compounds, none of which had known bioactivity, 20 were prioritized for further characterization based on features of their chemical structures. These compounds were denoted small molecule inhibitors of p27 depletion, SMIPs.

### Activity and specificity of SMIPs in p27 accumulation

Sixteen of the 20 SMIPs were obtainable as powders from commercial sources. The identity and integrity of all compounds was confirmed by high-performance liquid chromatography (HPLC) and mass spectrometry (data not shown). SMIPs were retested for activity in a dose-response experiment using the primary screening assay. Thirteen out of 16 purchased SMIPs induced a ≥ twofold increase in the percentage of p27 positive cells at the maximal dose with the remaining compounds having lower activity (data not shown). SMIP014 was identified as an additional false positive at this step (data not shown).

**Figure 4:**
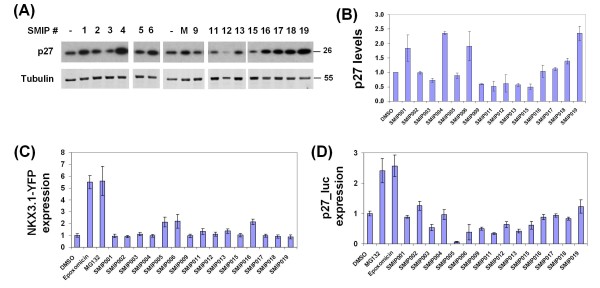
**Validation of prioritized small molecule inhibitors of p27 depletions (SMIPs)**. (a) Accumulation of p27 upon treatment with SMIPs. LNCaP-S14 cells were treated for 18 h with the respective SMIPs (40 μM), DMSO (-) or the positive control MG132 (M). The data is representative of three independent experiments. (b) Quantification of three independent immunoblotting experiments by film densitometry and normalization to the loading control tubulin. (c) Target pathway selectivity was evaluated in LNCaP cells stably transfected with NKX3.1-YFP. Cells were treated with 40 μM SMIPs for 18 h. YFP fluorescence was normalized to cell numbers and plotted relative to DMSO treated cells. The graph represents the mean +/- standard deviation (SD) from 16 replicates. (d) Cell type specificity was evaluated by measuring the accumulation of p27Luc in Hela_p27Luc cells. Luciferase activity was measured in total cell lysate from cells treated with 40 μM of the respective SMIPs. The graph is representative of three independent experiments and shows the mean +/- SD from eight replicate measurements. Proteasome inhibitors MG132 and epoxomycin were used as positive controls.

Several SMIPs also induced p27 accumulation as determined by immunoblotting (Figure 4a and 4b), although the effects were generally weaker (~twofold at 40 μM). While seemingly small, such an increase is biologically significant because p27 levels do not vary more than two- to threefold during a normal cell cycle ([40] and our own unpublished observation). This apparent discrepancy between the immunofluorescence and immunoblotting data, which we had encountered previously during assay development (with positive control compounds roscovitine and MG132; Figure 1c and data not shown), is explained by the different metrics of the assays: While the immunofluorescence assay determines the percentage of cells with nuclear p27 staining above a threshold determined by staining with secondary antibody alone, the immunoblotting assay measures the total amount of p27 that can be extracted from a cell population (no threshold).

Further prioritization of the remaining 15 SMIPs was achieved by counter screening against another unstable prostate tumour suppressor (NKX3.1) [41,42]. In order to address target specificity, LNCaP cells stably overexpressing NKX3.1 fused to yellow fluorescent protein (NKX3.1-YFP) were used. Cells were treated with a single concentration (40 μM) of SMIPs in 384-well plates and analysed by automated microscopy (see the Methods section). The Z' factors for this assay, as determined by positive and negative controls, were between 0.62 (epoxomycin) and 0.67 (MG132), while the signal-to-background ratio was five in both cases. Although some SMIPs (005, 006, 016) caused a ~twofold increase in the percentage of NKX3.1 positive cells, the effect was subtle in comparison to proteasome inhibitors (~fivefold; Figure 4c) and could not be confirmed by immunoblotting (data not shown). Likewise, when the expression of endogenous NKX3.1 was evaluated in DU145, another prostate cancer cell line with very low levels of NKX3.1, none of the SMIPs scored positive (data not shown). The minor effects of some SMIPs in the NKX3.1-YFP fluorescence assay were most likely an artifact of compound autofluorescence at the wavelength used to excite YFP.

We also determined the effect of SMIPs on a HeLa cell line stably expressing p27 fused to firefly luciferase (p27-luc) [40,43]. While proteasome inhibitors led to an increase in p27-luc expression, which was readily apparent by luciferase assay (Figure 4d) and immunoblotting (data not shown), none of the SMIPs were active. This finding suggests a certain degree of cell type specificity of SMIP action.

### SMIPs inhibit cell growth and induce cell death

Since p27 can drive cell cycle arrest, senescence, and apoptosis [7,44,45], we assessed the effect of SMIPs on cell proliferation and viability. LNCaP-S14 cells and IMR90 normal human fibroblasts were exposed to increasing concentrations of SMIPs for 72 h and scored using the MTT assay followed by calculation of IC_50 _values (concentration required for 50% inhibition) (Table 1). Whereas SMIP012 and 016 were moderately toxic in normal fibroblasts, SMIPs 001 and 004 showed substantial cancer cell specificity being at least five times more potent in LNCaP-S14 than in IMR90 cells. Although SMIP005 also showed good cell type selectivity, it was excluded at this point due to structural features that suggested potential unspecific reactivity with cellular macromolecules. Based on their apparent cancer cell specificity and their chemical structures, SMIPs 001 and 004 were selected for additional studies.

### SMIPs induce dose-dependent accumulation of p27 and p21

**Figure 5:**
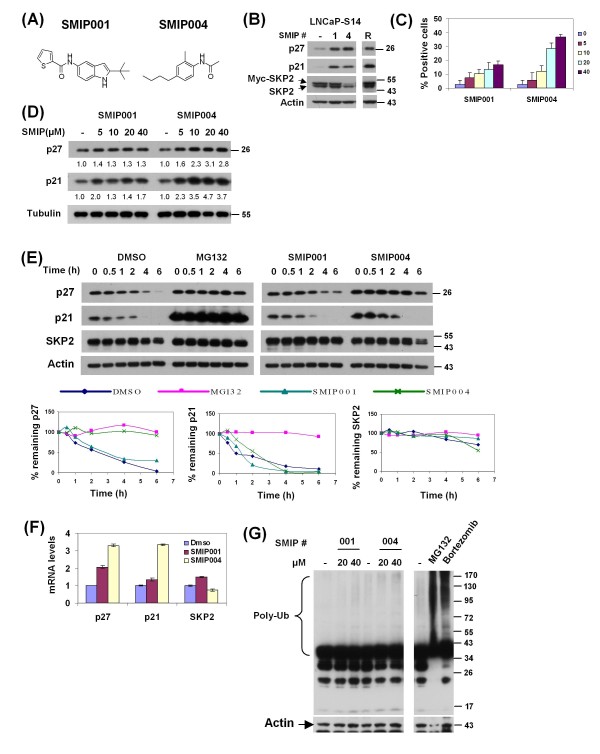
**Validation of SMIP001 and SMIP004**. (a) Chemical structure of the two small molecule inhibitors of p27 depletions (SMIPs) identified in the screen. (b) Cell lysates from LNCaP-S14 treated with 40 μM SMIPs for 24 h were analysed by immunoblotting. Roscovitine (R) was used as positive control. The data shown is representative of two independent experiments. (c) LNCaP-S14 cells were treated with increasing concentrations of SMIPs (5-40 μM) for 18 h followed by fixation and staining for p27. The graph represents data from 24 replicate wells per concentration. (d) LNCaP-S14 cells were treated with SMIPs (5-40 μM) for 18 h and analysed by immunoblotting for the levels of p27 and p21. (e) p27, p21 and SKP2 stability in LNCaP-S14 cells treated with 40 μM SMIPs for 18 h. Cycloheximide (CHX, 100 μg/mL) was added for the indicated time points prior to preparation of cell lysates and immunoblot analysis. MG132 was used as positive control. The graphs represent the quantification of p27, p21 and SKP2 levels relative to the zero hour time point. The data is representative of three independent experiments. (f) LNCaP-S14 cells were treated with SMIP00s (40 μM) for 18 h followed by extraction of total RNA. The mRNA levels of p27, p21 and SKP2 were quantified by quantitative polymerase chain reaction as described in the Methods section. All data was normalized to GAPDH and is expressed as fold induction. The graph is representative of two independent experiments. (g) Total lysates of LNCaP-S14 cells treated with DMSO (-) or SMIPs (20 and 40 μM) for 24 h were analysed by immunoblotting using anti-ubiquitin antibody. The proteasome inhibitors, MG132 (20 μM) and bortezomib (100 nM) were used as positive controls. The blot is representative of two independent experiments.

Figure 5a shows the chemical structures of the two compounds. We next confirmed the accumulation of p27 upon treatment with 40 μM SMIP001 and 004 in LNCaP-S14 cells (Figure 5b). The CKI p21, which was described as another ubiquitylation target of SKP2 [46], was also upregulated by SMIPs (Figure 5b). The effects on p27 and p21 were also observed with the CDK inhibitor roscovitine. Interestingly, SMIP004 caused a decrease in the levels of both the endogenous and the exogenous, stably expressed SKP2. In contrast, treatment of normal human fibroblasts with SMIPs did not affect p27 or p21 levels (see below).

In order to evaluate the dose-response of the effect on p27, LNCaP-S14 cells were treated with increasing concentrations of the respective SMIPs (5 - 40 μM), and the percentage of cells with nuclear p27 was measured by immunofluorescence and automated microscopy. Both SMIPs led to a dose-dependent increase in p27 positive cells (Figure 5c). Dose-dependent increases in p27 and p21 were also observed by immunoblotting (Figure 5d). Whereas SMIPs 004 led to ~threefold induction at 40 μM, SMIP001 was less active in this experiment causing only a modest accumulation of p27 (30%-40%).

In order to begin to determine the mode of action of SMIPs, we measured their effects on p27 protein stability in a cycloheximide chase experiment. Whereas the half-life of p27 was ~ 1.29 h in cells treated with DMSO, a value that is in good agreement with published reports (for example, [47]), treatment with either MG132 or SMIP004 increased p27 half-life to > 6 h (Figure 5f). No effect on p27 stability was seen for SMIP001. We also evaluated the stability of p21 and SKP2 but did not see any effects of SMIPs on these proteins. In summary, these data revealed a striking correlation between the ability of SMIP004 to downregulate SKP2 and to induce p27 stabilization. In contrast, SMIP001 appears to regulate p27 and p21 primarily at the level of mRNA expression (Figure 5f).

Since SMIP004 replicated the effect of MG132 on p27, we tested whether it acts as a proteasome inhibitor. Total cell lysate from LNCaP-S14 cells treated with 20 and 40 μM of the SMIPs were analysed by immunoblotting with anti-ubiquitin antibody to assess the abundance of polyubiquitylated proteins. None of the compounds caused the accumulation of polyubiquitylated proteins that was readily seen with the proteasome inhibitors MG132 and bortezomib (Figure 5g). Likewise, none of the SMIPs inhibited the proteolytic activity of purified 26S proteasomes toward fluorogenic peptide substrates even when assayed at 40 μM concentration (data not shown).

### SMIPs cause inhibition of cellular CDK2 activity, G1 delay and apoptosis in LNCaP-S14 cells

**Figure 6:**
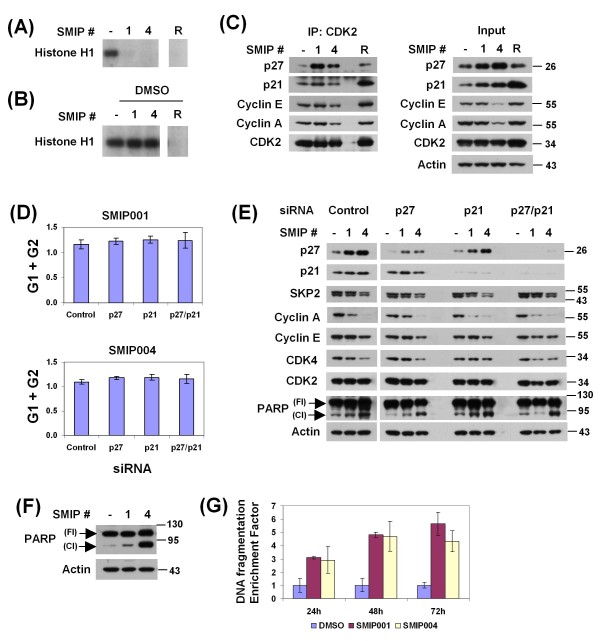
**Inhibition of CDK2 kinase activity and induction of apoptosis by small molecule inhibitors of p27 depletions (SMIPs)**. (a) Histone H1 kinase assays were performed with CDK2 complexes immunopurified from LNCaP-S14 cells treated with 40 μM SMIPs for 24 h as described in Methods. Roscovitine (R) was used as positive control. (b) Histone H1 kinase assays with CDK2 complexes immunopurified from dimethyl sulfoxide (DMSO) treated LNCaP-S14 cells. The indicated SMIPs (20 μM) were added to the reactions. Roscovitine (R) was used as a positive control for kinase inhibition *in vitro*. (c) Analysis of CDK2 complexes. Equal amounts of protein from LNCaP-S14 cells treated with DMSO, SMIPs (40 μM) or roscovitine (R, 20 μM) were immunoprecipitated using an anti-CDK2 antibody as described in the Methods section. The levels of co-precipitated cyclin E, cyclin A, p27, and p21 were evaluated by immunoblotting. (d) LNCaP-S14 cells were transfected with siRNAs for p27 and p21 as described in the Methods section and treated with DMSO or SMIPs (40 μM) for 24 h. The cell cycle distribution was analysed by flow cytometry. The graphs represent the population of SMIPs-treated cells in G1 and G2 normalized to DMSO-treated cells for each siRNA. (e) Total cell lysates from siRNA transfected cells were subjected to immunoblotting with the specified antibodies. The blots are representative of three independent experiments. (f) Apoptosis was evaluated in total cell lysates from LNCaP-S14 cells treated with 40 μM SMIPs for 24 h. Poly ADP ribosome polymerase (PARP) cleavage was visualized using an antibody that detects both full length (Fl) and cleaved (Cl) PARP. The blot is representative of three independent experiments. (g) Quantification of mono and oligonucleosomes as indication of apoptosis. Lysates of cells treated with DMSO or SMIPs (40 μM) for 24 h were obtained and analysed for cytoplasmic histone-associated DNA fragments as described in the Methods section. The graph represents the DNA fragmentation enrichment factor ± standard deviation of two independent experiments.

Since SMIPs robustly upregulated p27 and p21, we asked whether this resulted in G1 delay. Indeed, SMIPs 001 and 004 induced a G1 delay with a concomitant decrease in the S phase population (Table 2). We next asked whether the cell cycle delay coincided with inhibition of CDK2, one of the major cellular target kinases of p27 and p21. CDK2-cyclin complexes were immunoprecipitated from lysate of LNCaP-S14 cells treated with DMSO, the CDK inhibitor roscovitine or SMIPs and assayed for activity toward histone H1 *in vitro*. As with roscovitine, application of SMIPs led to strong inhibition of the CDK2 activity retrieved from cells (Figure 6a). In order to test the possibility that SMIPs act like roscovitine as direct inhibitors of CDK2 kinase activity, SMIPs were added to CDK2 complexes purified from untreated cells. Unlike roscovitine, none of the SMIPs inhibited CDK2 activity when added to the kinase reaction *in vitro *(Figure 6b), indicating that the two SMIPs are not kinase inhibitors.

**Table 2:** Cell cycle distribution in protein prostate cancer cell lines treated with 40 μM small molecule inhibitors of p27 depletion (SMIPs) for 24 and 48 h.

			LNCaP			LNCaP -S14			DU145			PC3			IMR90	
Time	SMIP	G1	S	G2	G1	S	G2	G1	S	G2	G1	S	G2	G1	S	G2
24 h	DMSO	70.8	18.0	10.9	64.8	23.3	10.6	40.5	44.0	15.3	42.2	26.7	30.0	67.2	16.6	16.2
	SMIP001	**84.5**	6.6	7.6	**84.8**	7.2	6.3	**65.6**	23.9	9.7	**48.6**	20.4	29.7	68.4	16.2	15.3
	SMIP004	**81.7**	7.9	9.8	**72.3**	19.1	7.1	**75.7**	14.1	8.0	**58.5**	14.3	25.7	68.6	18.2	13.2
48 h	DMSO	70.4	17.8	10.0	66.4	22.3	7.5	50.7	29.8	17.7	46.3	20.8	31.2	77.0	12.8	10.3
	SMIP001	**83.6**	8.5	6.6	**86.8**	6.9	3.5	**71.1**	13.4	13.6	**57.5**	13.6	27.8	77.0	8.9	14.1
	SMIP004	**87.3**	3.5	6.6	**75.8**	14.7	6.4	**79.7**	9.0	10.4	**65.8**	8.5	24.3	77.6	11.5	10.9

The data is representative of two independent experiments.

We also evaluated the effect of SMIPs on the levels of p27, p21 and cyclins E and A complexed with CDK2 by coimmunoprecipitation. Both SMIP001 and 004 led to a strong increase in the recruitment of p27 to CDK2, while SMIP001 also slightly increased coprecipitation of p21 (Figure 6c). SMIP004 also reduced the amounts of cyclins E and A retrieved with CDK2 (Figure 6c). This was paralleled by a marked downregulation of cyclins E and A upon SMIP004 treatment (Figure 6c). A more variable downregulation of cyclin A and CDK4 was observed with SMIP001 (Figure 6e). Taken together, these findings suggested that SMIP-induced inhibition of CDK2 activity might be a combined consequence of p27/p21 upregulation and cyclin E/A downregulation.

In order to determine the specific contribution of p27 and p21 to SMIP-induced cell cycle delay, we performed siRNA-mediated knockdown studies. The depletion of p21 and p27 led to a decrease in the G1 population of untreated cells by 6 - 13 percentage points (Additional File 2), a finding that indicates biologically significant effects at the knockdown efficiencies achieved (~80%, Figure 6e). Surprisingly, however, neither individual nor combined knockdown of the two CKIs was able to abrogate SMIP-induced cell cycle delay (Figure 6d and Additional File 2). Likewise, CDK2 activity in SMIP-treated cells was not rescued by knockdown of p27 and p21 (data not shown). Finally, neither the downregulation of cyclins E and A nor that of CDK4 was consistently affected by knocking down the CKIs, suggesting that these effects of SMIPs probably account primarily for their G1 delay activity.

In addition to G1 delay, treatment of LNCaP-S14 cells with SMIPs for 24 h caused apoptosis as determined by the cleavage of poly-ADP ribose polymerase (PARP, Figure 6f and 6g). The apoptotic effect of SMIPs was independently evaluated by measuring the amount of cytoplasmic histone-associated DNA fragments. Cells treated with 40 μM SMIP001 and 004 from 24 to 72 h showed a three- to fivefold increase in the amount of mono and oligonucleosomes, thus confirming the apoptosis-inducing activity of SMIPs.

### Effects of SMIPs in other prostate cancer cell lines

**Figure 7:**
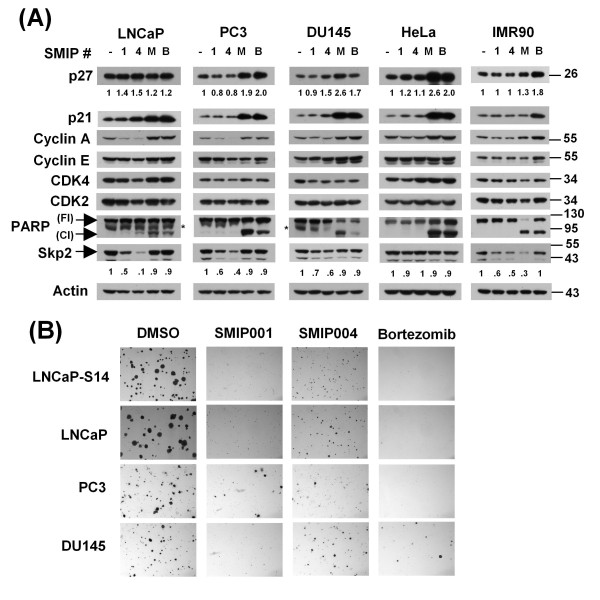
**Effects of small molecule inhibitors of p27 depletion (SMIPs) in prostate cancer cell lines**. (a) Prostate cancer cell lines, LNCaP, PC3 and DU145 as well as HeLa and IMR90 cells were treated with vehicle (dimethyl sulfoxide; DMSO), SMIPs (40 μM) or the proteasome inhibitors, MG132 (M, 20 μM) and bortezomib (B, 100 nM) for 24 h. The levels of the indicated proteins were evaluated by immunoblotting. Asterisks denote an unspecific band. (b) The indicated cell lines were grown in soft agar and treated with vehicle (DMSO), SMIPs (40 μM), or bortezomib (100 nM) with addition of fresh compounds in three-day intervals as described in the Methods section. The images are representative of two independent experiments.

As shown in Table 2, SMIPs also induced robust G1 delay in parental LNCaP cells as well as in two other prostate cancer cell lines, DU145 and PC3. No such effect was seen with HeLa cells or IMR90 fibroblasts. In parental LNCaP cells, G1 delay correlated with an increase in the levels of p27 and p21 as well as a downregulation of cyclins A and E and CDK4, which was particularly prominent with SMIP004 (Table 3 and Figure 7a). Unlike in SKP2 overexpressing LNCaP-S14 cells, SKP2 was downregulated by both SMIP001 and 004 in parental LNCaP cells, although the suppression was substantially greater with SMIP004 than 001 (Figure 7a). Less pronounced effects on SKP2 were also observed in PC3 and DU145 cells and in IMR90 fibroblasts. In contrast, when averaged over four independent experiments, SMIPs had no consistent effect on p27 levels in PC3 and DU145 cells, although a minor increase in p21 levels was observed (Table 3) that coincided with a modest reduction in the levels of cyclin A and CDK4 (Figure 7a). None of the latter effects were apparent either in HeLa or IMR90 cells. Despite a lack of induction of apoptosis (PARP cleavage, Figure 7a) or overt cytotoxicity (Table 4), the SMIP-induced G1 delay of PC3 or DU145 cells resulted in the same robust inhibition of colony formation in soft agar as observed in LNCaP-S14 and parental LNCaP cells (Figure 7b).

**Table 3:** Quantification of p27 and p21 protein levels in protein prostate cancer cell lines, HeLa and IMR90 cells treated with 40 μM small molecule inhibitors of p27 depletion (SMIPs) for 24 h.

	LNCaP-S14		LNCaP		PC3		DU145		HeLa		IMR90	
SMIP	p27	p21	p27	p21	p27	p21	p27	p21	p27	p21	p27	p21
DMSO	1.0 ± 0.0	1.0 ± 0.0	1.0 ± 0.0	1.0 ± 0.0	1.0 ± 0.0	1.0 ± 0.0	1.0 ± 0.0	1.0 ± 0.0	1.0 ± 0.0	1.0 ± 0.0	1.0 ± 0.0	1.0 ± 0.0
SMIP001	**1.9 ± 0.5**	**2.0 ± 0.6**	**1.5 ± 0.7**	**1.7 ± 0.6**	1.3 ± 0.6	1.1 ± 0.5	1.1 ± 0.2	1.1 ± 0.3	1.1 ± 0.1	1.1 ± 0.2	1.0 ± 0.0	1.1 ± 0.0
SMIP004	**2.4 ± 0.7**	**2.8 ± 0.7**	**2.1 ± 1.1**	**2.7 ± 0.1**	1.1 ± 0.3	**1.7 ± 0.9**	1.3 ± 0.1	**1.7 ± 0.5**	1.1 ± 0.2	1.1 ± 0.1	1.2 ± 0.3	1.1 ± 0.0
MG132	1.5		1.9 ± 0.1	2.9	2.0 ± 1.0	4.1 ± 0.4	2.4 ± 0.1	3.1	2.2 ± 0.9	5.9 ± 1.7	0.9 ± 0.5	1.1 ± 0. 1
Bortezomib	1.8		2.1		2.5 ± 1.0	4.7 ± 1.4	1.7 ± 0.5	2.5 ± 0.4	1.3 ± 0.2	2.2 ± 0.8	1.8 ± 0.0	1.8 ± 0.4

The data is representative of at least four independent immunoblotting experiments.

**Table 4:** Cytotoxicity was assessed by MTT assay in prostate cancer cell lines and normal fibroblasts treated with increasing concentrations (0.01 - 80 μM) of the respective small molecule inhibitors of p27 depletion (SMIPs) for 72 h and 96 h.

	SMIP001		SMIP004	
Cell line	72 h	96 h	72 h	96 h
LNCaP-WT	47.1	32.7	40	2.4
PC3	~ 80	80	NE	NE
DU145	~ 40	32.5	NE	NE
IMR90	40-80	40-80	NE	NE

NE: No effect.

The data in the table represents the IC50 (μM). NE: No effect.

## Discussion

The intimate link between p27 depletion and cancers deriving from the prostate and many other tissues renders pathways controlling p27 abundance attractive targets for the development of novel cancer therapeutics [48]. At the same time, the complexity and apparent redundancy of p27 regulatory pathways raises doubts as to whether targeting a single enzyme or proximal regulator can lead to sustained p27 accumulation in tumour cells. Rather than screening for inhibitors of SCF^SKP2 ^or other enzymes controlling p27, we have developed a cell-based phenotypic screening system to identify compounds that can modulate the p27 regulatory network in cancer cells such that normal nuclear p27 levels are restored. The platform was validated in a pilot screen that identified two compounds of previously unknown biological activity that efficiently reversed the depletion of p27 in prostate cancer cells at low micromolar levels. One of these compounds, SMIP004, increased p27 levels by prolonging the half-life of the p27 protein. Therefore, p27 depletion can be reversed by small molecules in cancer cells overexpressing SKP2.

Whereas the cell-based format readily revealed compounds that robustly modulated the screening endpoint - nuclear p27 levels - in intact cells, our pilot screen also exposed the disadvantages of this approach, namely that the molecular targets of SMIPs remain unknown and that the modulated screening endpoint is not necessarily causal to the ultimate cellular effects of the compounds identified. For example, our RNA interference studies showed that neither p27 nor p21 were required for SMIP-induced G1 delay in LNCaP-S14 cells. It is unlikely that inefficiency of the small interfering RNA (siRNA)-mediated knockdown obscured such a requirement for SMIP action, since depletion of p21 and p27 decreased the fraction of cells in G1 thus indicating that the knockdown efficiencies achieved here were functionally consequential. In addition, p21 and p27 levels in SMIP-treated knockdown cells were still lower than or equal to the levels in untreated control cells, suggesting that the minor accumulation of the CKIs observed upon SMIP administration to knockdown cells will be insufficient to cause a cell cycle arrest.

Two potential explanations can be offered to rationalize the dispensability of p21 and p27 for SMIP-induced G1 delay induced by SMIPs: First, it has been well established that mouse embryonic fibroblasts lacking p27 and p21 remain proficient in responding to negative proliferation signals with cell cycle arrest because the pocket proteins p107 and p130, which have CKI activity themselves, compensate for the loss of the p27/p21 [49,50]. Secondly, SMIPs induced downregulation of various positive cell cycle regulators, including cyclins E and A and CDK4, which was fully maintained upon p27 and p21 knockdown. Thus, it is conceivable that the combined effect of cyclin/CDK downregulation and CDK inhibition by pocket proteins and other CKIs accounts for SMIP-mediated cell cycle delay in the absence of p27 and p21.

Although it appears that the compounds identified in the present pilot screen caused p27 upregulation as a secondary consequence of cell cycle delay, using nuclear p27 accumulation as a screening endpoint readily enabled the identification of cell permeable compounds with antiproliferative activity. Notably, both SMIPs show cancer cell selectivity as they induce cell cycle delay and/or apoptosis in four different prostate cancer cells but not in normal human fibroblasts. Moreover, they inhibit colony formation in soft agar, which is a hallmark of the transformed phenotype of cancer cells and considered a stringent surrogate for *in vivo *tumour formation [51,52]. Finally, when applied to a more diverse set of compounds with refined drug-like structures, our screening platform may lead to the identification of more potent and direct modulators of nuclear p27 accumulation, similar in characteristics to the recently identified proteasome inhibitor argyrin A, which induces cell cycle arrest and/or apoptosis that is strictly dependent on p27 [45].

The high content screening platform described here also enables chemical genetics strategies for the identification of novel cellular pathways and targets impinging on p27 - for example, the observation that SMIP004 strongly downregulates SKP2 appears significant with regards to its mechanism of action. In a previous work we demonstrated that SKP2 is a rate-limiting component for p27 degradation in LNCaP cells [33]. A recent study in mice revealed that inactivation of SKP2 induces tumour cell senescence that is dependent upon p27, p21 and the transcription factor ATF4 [53], which is an integral component of the unfolded protein response (UPR) that is activated in response to endoplasmic reticulum stress. Although the exact mechanism by which SMIP004 downregulates SKP2 is presently unknown, we have observed a robust activation of UPR signalling by the compound (ERB and DAW, manuscript in preperation). A similar downregulation of SKP2 has been noticed with the proteasome inhibitor bortezomib, which is also an UPR inducer [54]. Mechanism of action studies on SMIP004 are therefore beginning to suggest a link between UPR-mediated downregulation of SKP2 and the accumulation of p27 (and perhaps p21). However, a substantial future effort will be required to decipher the exact molecular targets of the compounds identified here. Once those are revealed, target directed high throughput screening campaigns could be initiated to identify a more diverse set of compounds with improved potency toward p27 and clinical potential.

## Conclusions

The results shown here provide proof-of-principle that the cell-based screen we developed provides an effective means of identifying bioactive molecules with cancer selective antiproliferative activity. The advantage of a cell-based screening format is, however, offset by the limitation that the modulated endpoint is not necessarily causal to the ultimate cellular effects of identified compounds, thus requiring additional pathway deconvolution studies. Nevertheless, this approach can be applied to larger and more diverse sets of compounds with refined drug-like properties, revealing both unknown cellular pathways globally impinging on p27 and novel chemotherapeutic lead agents.

## Methods

### Compound libraries

NINDS, Prestwick Peakdale 1, Mixed Commercials, Antimitotic, and Enamine and Known Bioactive Compounds libraries were obtained from The Institute of Chemistry and Cell Biology (ICCB) at Harvard Medical School (MA, USA). Information about the libraries can be found at http://iccb.med.harvard.edu.

### SMIPs and other chemical compounds

SMIP001 to SMIP018 were purchased from Ryan Scientific (SC, USA). SMIP0019 was purchased from ChemDiv, Inc (CA, USA). Roscovitine was purchased from Enxo Life Sciences (formerly Biomol International; NY, USA), bortezomib from LC Laboratories (MA, USA), cycloheximide (CHX) and camptothecin from Sigma (MO, USA), MG132 and epoxomicin from BostonBiochem (MA, USA). All drugs were dissolved in DMSO and kept at -80°C.

### Antibodies

Anti-p27 antibody (610241, 1:5000 for immunoblotting and 1:1000 for immunofluorescence) was obtained from BD Biosciences (NJ, USA). Anti-SKP2 (32-3300, 1:2000 for immunoblotting.) and anti-ubiquitin (13-1600, 1:2000) was from Zymed Laboratories (CA, USA). Anti-SKP2 (sc-7164, 1:500 for immunofluorescence), anti-CDK2 (sc-163, 1:1000 for immunoblotting, 2 μg/500 mg protein for immunoprecipitation) and anti-CDK4 (sc-260, dil 1:500) from Santa Cruz Biotechnology, Inc (CA, USA). Antibodies for Cyclin E Ab-2 (clone HE12, dil 1:200) and Cyclin A Ab-6 (clone 6E6, dil 1:100) from Thermo Scientific (CA, USA). Anti-p21 (2947, 1:2000) and anti-PARP (9542, 1:1000) were from Cell Signaling Technology (MA, USA). Anti-Tubulin (T5168, 1:5000) was from Sigma Life Science. Anti-Actin (69100, 1:20000) was from MP Biomedicals, LLC (CA, USA). HRP Donkey anti-mouse IgG (715-035-150, 1:5000) and HRP Donkey anti-rabbit IgG (711-035-152, 1:5000) was obtained from Jackson ImmunoResearch Laboratories (PA, USA).

### Creation of the LNCaP-S14 cell line and culture conditions

Parental LNCaP cells were stably transfected with a plasmid driving the expression of Myc epitope-tagged SKP2 or empty vector (pcDNA3). Briefly, 5 x10^6 ^cells were transfected with 20 μg of each plasmid using Lipofectamine 2000 reagent (Invitrogen, CA, USA) following the manufacturer's protocol. Cells were selected in 600 μg/mL geneticin (G418) until individual colonies were visible. Colonies were picked, passed to 24 well plates and expanded. The levels of p27 and SKP2 were analysed by immunoblotting as described below. LNCaP-S14 (positive clone for SKP2 overexpression), LNCaP-P10 (vector control cell line), LNCaP (parental cell line), PC3 and DU145 cells were maintained in RPMI supplemented with 10% fetal bovine serum and 5% penicillin/streptomycin solution. IMR90 and HeLa cells were maintained in DMEM containing 10% fetal bovine serum, 5% penicillin/streptomycin and 4 mM glutamine. Cells were grown as a monolayer in a humidified incubator at 37°C and 5% CO_2_.

### Immunofluorescence assay on cover slips and in 384 well plates

The screen relies on the detection of differences in the levels of endogenous p27 by immunofluorescence. LNCaP-S14 cells were seeded at a density of 100,000 cells onto 15 mm glass cover slips and allowed to attach for 1 day. Cells were treated with vehicle (DMSO) or positive controls (roscovitine, MG132) for 18 h. After the incubation, cells were fixed by the addition of 10% paraformaldehyde in phosphate buffered saline (PBS; final concentration 4%) and incubated for 20 min at room temperature (RT). Cells were permeabilized by washing 3 × 5 min with PBS, 0.1% Triton X100, followed by blocking in 5% nonfat dry milk in PBS, 0.1% Triton X100 for 1 h at RT. Cells were incubated with 50 μl primary antibody (anti-p27, 1:1000 in blocking buffer) for 1 h, followed by one wash with blocking buffer. 50 μL of secondary antibody (Alexa Fluor 594 goat anti-mouse, Invitrogen A11005, 1:200 in blocking buffer) was added for 1 h. Cells were washed 3 × 5 min in PBS, 0.1% Triton X100 and stained with Hoechst dye (1 μg/mL in PBS) for 2 min. Cells were washed twice in PBS and mounted for imaging with a Nikon Type 120 inverted fluorescent microscope using 60× magnification. Compounds used as positive controls included MG132, epoxomycin and roscovitine, while DMSO was used as negative control. Cells stained with secondary antibody only were used to assess the non-specific (NS) staining background.

The above protocol was adapted to 384 well plates as follows: 4000 LNCaP-S14 cells per well were seeded into 384-well plates (black with clear bottom) in 30 μL RPMI, 10% fetal bovine serum (FBS). Nuclear p27 staining was done under the same conditions as above but with reducing the volume of solutions to 20 μL/well for 10% para-formaldehyde in PBS, 30 μL/well for blocking and wash solutions and 15 μL/well for primary and secondary antibody solutions and Hoechst dye. All liquid handling was done with an eight-channel multidrop liquid dispenser (Wellmate, OH, USA) and a wand aspirator (VP Scientific, CA, USA; VP-186L). After staining, plates were sealed and stored in the dark at 4°C until scanning.

### Images acquisition and analysis

Images from 384 well plates were acquired by using the ImageXpress Micro inverted epifluorescent automated microscope (Molecular Devices, CA, USA) at dual wave length to detect Hoechst and p27. The microscope was equipped with the Photometrics CoolSNAP ES digital CCD camera and an automated objective and filter cube changers. Two images per well at a 20× magnification were obtained at each wave length.

Images were analysed with the MetaXpress cellular imaging analysis software using the cell scoring module. Cells were scored positive for Hoechst, if the integrated pixel intensity was 210-fold above local background and positive for p27 when staining was 30-fold above background (signal obtained with secondary antibody). MetaXpress processing of the raw images provided quantitative measures of the total cell number and the number of p27 positive cells in a given field. The data from both images of each well were combined to get a single number of positive cells. The percentage of positive cells was calculated relative to the total number of cells. Background correction was done by subtracting the number of p27 positive cells in wells stained with secondary antibody only.

The staining protocol was also evaluated using another imaging and software package, the Cell Lab IC100 and Cytoshop software (Beckman Coulter, CA, USA). Minor changes were introduced to the protocol: The cell number was reduced to 3000/well and the secondary Alexa Fluor 568 goat anti mouse antibody was diluted 1:500 in blocking buffer. Four images per well were taken using 10× magnification with a numerical aperture of 0.25 and a camera binning of 2 × 2. A manual threshold was established by comparison of the total nuclear intensity in the positive control and the total intensity obtained in cells stained with the secondary antibody only (unspecific staining) and/or the vehicle control (DMSO). Processing of the raw images provided quantitative measures of the total cell number and the number of p27 positive cells in a given field.

Z'-factor calculation: This parameter was used to assess the quality of the assay in the HTP optimization (HTP Company, CA, USA) [37]. In three independent experiments, LNCaP-S14 cells were seeded in 384 well plates followed by treatment with 0.3% DMSO (192 wells, negative control) or 20 μM roscovitine (192 wells, positive control) for 18 h. The percentage of p27 positive cells was determined as described above and the Z' factor calculated from 576 replicates (each condition) as follows: Z'= 1- (3SD_+ _+ 3SD_-_)/(Ave_+ _- Ave_-_), where SD_+ _is the standard deviation for the positive control, SD_- _the standard deviation for the negative control, Ave_+ _the average for the positive control and Ave_- _is the average for the negative control [37].

### Primary screening

The cell-based screen was performed at the Institute of Chemistry and Cell Biology, Harvard Medical School, using libraries of uncharacterized compounds (Peakdale 1, Mixed Commercials, Antimitotic and Enamine, total of 4888 compounds) plated in 5 mg/mL stock solutions in DMSO, the NINDS library (1040 compounds, 10 mM in DMSO), the Prestwick Library (960 compounds, 2 mg/mL) and a library of 489 known bioactive compounds (plated at 5 mg/mL, 1.1 mg/mL, and 0.25 mg/mL in DMSO) for a total of 7638 compounds. LNCaP-S14 cells were seeded onto 384 well plates as described above. Compound stocks (100 nL) were pin-transferred using a robot-controlled stainless-steel pin array, resulting in a 300-fold-dilution (5 to 35 μM final screening concentration, depending on individual compound molecular weight) followed by incubation for 18 h. All compound plates were screened in duplicates. Each individual plate contained at least eight positive controls (roscovitine, 20 μM), eight negative controls (DMSO, 0.3%) and eight wells of cells treated with 0.3% DMSO and stained only with secondary antibody (non-specific staining). Staining of p27, imaging and analysis was performed as described above. Potential hits were classified according to their Z score. The Z score is a simple and widely known normalization method calculated as follows: Z = X_i _- X/S_x_, where X_i _is the raw measurement on the ith compound, X and S_x _are the mean and the standard deviation, respectively, of all measurements within each plate [38]. Potential hits were classified as weak if their Z score was between 2 and 3, medium if the Z score was between 3 and 5 and strong if the Z score was greater than 5.

### Counter screening

Target specificity was analysed using LNCaP cells stably expressing the homeobox transcription factor NKX3.1 fused to YFP. In brief, 4000 LNCaP-NKX3.1-YFP (clone 4A) cells were seeded in a 384 well plate and treated with SMIPs for 18 h. Proteasome inhibitors were used as positive controls. Images from 384 well plates were taken using the Cell Lab IC100 automated microscope (Beckman Coulter), and the percentage of cells positive for NKX3.1-YFP was quantified using Cytoshop software (Beckman Coulter). The percentage of NKX3.1-YFP positive cells was determined relative to DMSO treated cells.

Cell specificity was evaluated using HeLa cells stably transfected with a p27-luciferase (p27Luc) construct [43]. Briefly, HeLa-p27Luc cells were seeded in 96 well plates and treated with 40 μM of the respective SMIPs or positive controls (proteasome inhibitors). After incubation for 18 h, cells were lysed using Cell Culture Lysis Reagent from Promega (WI, USA) and luciferase activity was determined by the Luciferase Assay System (Promega) using a Veritas microplate luminometer (Turner Biosystems, CA, USA). Protein concentration was measured in a parallel plate using Bio-Rad Protein Assay (Bio-Rad, CA, USA). Luciferase activity was normalized against protein concentration and compared to the activity recovered from DMSO-treated cells.

### Immunoblotting

Total cell lysate from cells treated with vehicle (DMSO) or compounds were obtained by incubating cells in lysis buffer (25 mM Tris-HCl pH 7.4, 150 mM NaCl, 0.5% Triton X-100, protease inhibitors) for 15 min at 4°C. The supernatant was collected by centrifugation and the amount of protein was determined with a Bio-Rad protein assay (Bio-Rad) using BSA as standard. Equal amounts of protein were subjected to SDS-PAGE (4%-20% gels, Invitrogen) and transferred to PVDF membranes. The membranes were blocked with TBST (50 mM Tris-HCl pH 7.5, 150 mM NaCl, 0.05% Tween-20) containing 5% non-fat dry milk or bovine serum albumin (BSA) for 1 h at room temperature, followed by incubation in primary antibodies 4°C overnight. Membranes were washed and incubated with secondary antibodies conjugated to HRP (Jackson ImmunoResearch Laboratories) for 1 h at room temperature. Signals were visualized using the ECL Western Blotting Substrate (Pierce Biotechnology, IL, USA) according to the manufacturer's instructions. Membranes were stripped using Restore™ Western Blot Stripping Buffer (Pierce Biotechnology). Films were scanned and quantification of the bands was performed using Image J software http://rsbweb.nih.gov/ij/.

### Co-immunoprecipitation

Total lysates of cells treated with vehicle (DMSO), SMIPs or positive controls were prepared by incubation in immunoprecipitation (IP) buffer (25 mM Tris-HCl, pH 7.4; 150 mM NaCl, 0.5% Triton X-100, protease inhibitors) for 15 min at 4°C. Upon centrifugation, the supernatant was collected and the amount of protein was determined with a Bio-Rad protein assay (Bio-Rad Laboratories) using BSA as standard. Five hundred micrograms of protein was adjusted to 1 mL with IP buffer and incubated with 10 μL anti-CDK2 antibody (sc-163, Santa Cruz Biotech) at 4°C for 3 h with constant rotation, followed by addition of 100 μL protein G-sepharose beads (10% slurry) for 2 h. After four washes for 5 min each with 1 mL IP buffer, beads were resuspended in 2X-SDS Laemmli buffer, followed by SDS-PAGE and immunoblotting.

### *In vitro *kinase assay

Histone H1 kinase assays were performed as described in reference [55]. Briefly, the total cell lysates from LNCaP-S14 cells treated with the respective SMIP (40 μM) for 24 h were prepared in IP lysis buffer supplemented with protease inhibitors followed by IP. Kinase reactions were performed by adding histone H1 (1 μg) and 7.5 μCi [γ-^32^P]ATP (800 Ci/mmol Perkin Elmer Life Sciences, MA, USA) in kinase buffer (20 mM MgCl_2_, 10 mM EGTA, 40 mM Hepes, pH 7). After incubation at 30°C for 20 min, the reaction was stopped by adding 20 μL 2× SDS gel loading buffer. Samples were separated by electrophoresis, gels were stained and dried, followed by exposure to X-ray film. DMSO was used as negative control and roscovitine (CDK2 inhibitor) as positive control.

### Cytotoxicity assay

LNCaP-S14 (10,000 cells), PC3, DU145 or IMR90 cells (5000 cells) were seeded in 96-well plates and treated with increasing concentrations of the respective SMIPs for 72 h. Cell viability was assessed using the MTT cell proliferation assay (ATCC^®, ^Middlesex, UK) according to the manufacturer's protocol. IC_50 _was calculated using the BioDataFit 1.02 software http://www.changbioscience.com/stat/ec50.html.

### Determination of protein half-lives by cycloheximide chase

LNCaP-S14 cells were treated with SMIPs (40 μM) for 18 h followed by the addition of 100 μg/mL cycloheximide (CHX). Cell extracts were obtained as described above at different times after CHX addition. Protein lysate was subjected to SDS-PAGE and immunoblotting for p27, p21 and SKP2. Tubulin was used as loading control. Protein half-lives were calculated using a web based calculator http://www.1728.com/halflife.htm.

### RNA isolation and quantitative polymerase chain reaction (q-PCR)

Total RNA was isolated from LNCaP-S14 cells treated with 40 μM SMIPs or vehicle (18 h) using the RNeasy Mini Kit (Quiagen, CA, USA), followed by first-strand cDNA synthesis using Omniscript^® ^Reverse Transcription (Quiagen). Real-time PCR analysis was performed on the Stratagene Mx3000p detection system (Stratagene, Agilent, CA, USA) using the SYBR Green PCR Master Mix (ABI, CA, USA) at the Microarray and q-PCR Facility of the Sanford-Burnham Medical Research Institute. The primers used were:

5'-ACTGCAGAGACATGGAAGAG-3' and 5'-ATGCGTGTCCTCAGAGTTAG-3' for p27 (to amplify a region from nucleotides 608 to 852); 5'-CGATGGAACTTCGACTTTG-3' and 5'-GGGTACAAGACAGTGACAGG-3' for p21 (to amplify a region from nucleotides 142 to 355); and 5'-TGAGCTGCTAAAGGTCTCTG-3' and 5'-ATGTGCTGTACACGAAAAGG-3' for SKP2 (to amplify a region spanning from nucleotide 527 in exon No. 2 to 739 in exon No.4). Briefly, reactions were done in a 25 μL reaction volume of q-PCR mixture containing 2 μL of cDNA (40 ng) and 250 nM of each primer. Activation of the enzyme was done at 95°C for 10 min followed by 40 cycles of amplification at 95°C for 30 s, 56°C for 1 min and 72°C for 30 s. All reactions were done in duplicates and normalized using GAPDH as control. Primers were design using Primer 3 http://frodo.wi.mit.edu/primer3/ and synthesized by Valuegene, Inc, CA, USA.

### Cell cycle analysis

Cells were exposed to SMIPs (40 μM) for 24 h and 48 h. Cells were trypsinized, washed with PBS and suspended in 600 μL PBS. Cells were fixed by adding 1.4 mL cold absolute ethanol and kept at -20°C for at least 12 h. After washing once with PBS, the cells were resuspended in 250 μL PBS containing 2.5 μg RNAse A and incubated for 45 min at room temperature. Staining was done by adding 40 μg/mL of propidium iodine followed by incubation for 15 min at room temperature. DNA-bound fluorescence was read at 564 to 606 nm using FACSort and FACSCanto flow cytometers (BD Biosciences) at the Flow Cytometry Facility of the Sanford-Burnham Medical Research Institute. Distribution of cells in the different cell cycle phase was determined with ModFit LT 3.2.1 or FlowJo 8.6 software. Aggregates were excluded from the analysis manually using pulse shape or identified by automated modelling in ModFit LT.

### Apoptosis assay

Apoptosis was measured using the Cell Death Detection ELISA (Roche, Basel, Switzerland) following the manufacturer's instructions. Briefly, LNCaP-S14 cells (0.1 × 10^6^) were seeded in 12-well plates and treated with SMIPs (40 μM) for 24 h. Cells were collected in medium, spun at 1000 rpm, and resuspended in 1 mL PBS at the specified time points (day 1 to day 3). The cell suspension was divided into two parts. The first half was used for determination of cytoplasmic histone-associated DNA fragments (ELISA), the second for protein determination to normalize the ELISA data to the amount of input protein.

### siRNA transfection

LNCaP-S14 cells were seeded in 6 cm dishes or six-well-plates coated with poly-lysine the day before transfection. 10-20 nM of siRNA was transfected using DharmaFECT 3 (Thermo Scientific) according to the manufacturer's instructions. Briefly, siRNAs were dissolved in siRNA suspension buffer (Quiagen) at 20 μM, heated to 90°C for 1 min and incubated at 37°C for 1 h. siRNAs were added to the DharmaFECT 3 reagent diluted in Optimem media (1:100) and incubated for 20 min at room temperature. The mix was added to the cells for 24 h. Treatment with SMIPs (40 μM) or vehicle was carried on for another 24 h. Cells were obtained for FACS or lysed for immunoblotting analysis as described above. Untransfected cells as well as cells transfected with non-specific siRNA were used as controls. Silencer^® ^Negative Control siRNA No.1 from Ambion (Applied Biosciences, CA, USA) was used as non-specific control siRNA. siRNA target sequences for p27 and p21 synthesized by IDT (Integrated DNA Technologies Inc, Iowa, USA) were as follows; p27 siRNA: AAGGUUGCAUACUGAGCCAAG, and p21 siRNA: AACAUACUGGCCUGGACUGUU [56].

### Soft agar assay

Agar Noble (Difco, MD, USA) was suspended at 6% in water and autoclaved. A dilution 1:10 was made with RPMI culture medium and added to six-well plates (2 mL). We suspended 20,000 cells were in 0.5 mL RPMI culture medium (containing drugs), added to 0.5 mL of agar (0.6%) and poured immediately into a six-well plate containing hardened bottom agar. Cells were fed with fresh medium containing DMSO, SMIP001, SMIP004 or bortezomib every third days. Images were taken after 14 days using a Nikon Eclipse E600 Microscope.

## Abbreviations

ADT: androgen deprivation therapy; CDK: cyclin-dependent kinase; CHX: cycloheximide; DMSO: dimethyl sulfoxide; IB: immunoblotting; IP: immunoprecipitation; PCa: prostate cancer; q-PCR: quantitative polymerase chain reaction; siRNA: small interfering RNA; SMILES: simplified molecular input line entry specification; SMIP: small molecule inhibitors of p27 depletion; UPR: unfolded protein response; UPS: ubiquitin proteasome system; YFP: yellow fluorescent protein.

## Authors' contributions

The vast majority of the experiments in this study were carried out by the principal author, ERB. She developed the screening assay, performed all screening and counter screening, carried out the bulk of the validation assays and drafted the manuscript. CCY generated the LNCaP-NKX3.1-YFP cell line used for counter screening and assisted with advice. LL generated and characterized the LNCaP-S14 cell line used for screening. GPR assisted with advice in prioritizing SMIPs for follow-up based in their chemical features and directed the HPLC analysis of SMIPs. DAW developed the concept for this work, participated in study design, secured funding, assisted with kinase assays and edited the manuscript.

## Supplementary Material

Additional file 1 **Supplementary Table 1**. The table shows 176 small molecule inhibitors of p27 depletion (SMIPs) identified in the primary screening. Compounds were classified according to their Z factors as strong (S), medium (M) or weak (W). Their position in the screening (stock plate and well), simplified molecular input line entry specification (SMILES), name and vendor are also listed. A column with known biological actions is also shown for some compounds. Click here for file

Additional file 2 **Supplementary Table 2:** The table shows the cell cycle distribution of LNCaP-S14 transfected with small interfering RNA for p27 and p21 and treated with SMIP004 (40 μM) as described in the Methods section. Cell were analysed by flow cytometry and the percentage of cells in the different phases of the cell cycle were calculated. Click here for file
